# Distinct roles of class I PI3K isoforms in multiple myeloma cell survival and dissemination

**DOI:** 10.1038/bcj.2014.24

**Published:** 2014-04-25

**Authors:** I Sahin, M Moschetta, Y Mishima, S V Glavey, B Tsang, F Azab, S Manier, Y Zhang, P Maiso, A Sacco, A K Azab, A M Roccaro, I M Ghobrial

**Affiliations:** 1 Department of Medical Oncology, Dana-Farber Cancer Institute, Harvard Medical School, Boston, MA, USA; 2 Cancer Biology Division, Department of Radiation Oncology, School of Medicine, Washington University in St Louis, St Louis, MO, USA

The phosphoinositide 3-kinase (PI3K) pathway has a crucial role in tumor progression and drug resistance, including both conventional chemotherapeutics as well as novel agents.^1^ Although no mutations have been described in the PI3K/Akt genes in multiple myeloma (MM), it was shown that this pathway is constitutively activated in MM cells and has pleiotropic effects influencing proliferation, drug resistance, angiogenesis and cell adhesion.^2^

PI3Ks are divided into three subclasses, and of these, class I PI3Ks—p110α (also known as PIK3CA), p110β (also known as PIK3CB), p110γ (also known as PIK3CG) and p110δ (also known as PIK3CD)—are well described in terms of their role in cancer development and progression.^1, 3^ PIK3CA is frequently mutated in solid tumors including carcinoma of the prostate, breast colon and endometrium.^4, 5^ However, there have been no reports of cancer-specific mutations in MM.^6^

Recently, a number of potential therapeutics targeting specific PI3K groups or isoforms were developed.^3, 4^ Previous studies have indicated that p110α, p110β and p110δ might be potential targets for MM.^7, 8, 9^ Although the basic framework of PI3K signaling has been uncovered, the contribution of the different PI3K isoforms is not well understood.^4^ In the current study, we investigated the functional role of class I PI3K isoforms in modulating MM cell trafficking *in vivo* and *in vitro.*

To examine activation of the PI3K/Akt pathway in MM, we first performed gene set enrichment analysis^10^ on the gene-expression data set (Shaughnessy *et al.* ref. GSE24080) of patients in different International Staging System stages of MM compared with normal donors;^11^ and found enrichment of genes related to class I PI3K-activated AKT signaling events. These findings were observed in stage I, II and III MM patients compared with healthy individuals (Figure 1a).

To study the role of each isoform (p110α, β, γ, and δ) in regulating MM cell survival and trafficking *in vivo* and *in vitro*, the expression of PI3K isoforms was examined in a panel of eight MM cell lines showing different levels of expression of PI3K isoforms with only MM.1S expressing all isoforms (Figure 1b). Thus, MM.1S-GFP^+^/luc^+^ was infected with lentivirus-mediated small hairpin RNAs targeting the different PI3K isoforms. Stable cell lines were generated, and efficiency of knockdown for each isoform was confirmed by reverse transcription quantitative PCR (Figure 1c). Specificity of knockdown was demonstrated by immunoblotting in cell lines using specific antibodies against each isoform (Figure 1d). Then, we evaluated the effect of each isoform on PI3K–Akt signaling in MM cells in the context of primary MM bone marrow mesenchymal stromal cells (BM-MSCs) and found inhibition of BM-MSC-dependent induction of phospho(p)-Akt in MM cells with all PI3K isoforms silenced in the tumor clone (Figure 1e). Although p110α, β, and δ showed a modest reduction in cell survival *in vitro* (Figure 1f), cell cycle analysis revealed no significant difference on cell cycle distribution patterns (Supplementary Figure 1). We next performed adhesion assay of MM cells to primary MM-derived BM-MSCs; and found that by silencing each of class I PI3K isoforms, MM cells inhibited their adhesion properties, with the p110β and p110δ knockdown being the most effective (53% reduction and 47% reduction, respectively; *P*<0.001, *P*<0.01; Figure 1g).

To test the effect of the different p110 isoforms on MM tumor progression *in vivo*, SCID-Bg mice were injected with MM cells silenced for p110α, β, γ and δ, and tumor development was monitored by bioluminescence imaging. Scramble-infected cells were used as control. In consistent with *in vitro* data demonstrating that the most significant changes were observed for adhesion of MM cells to BM-MSCs in p110β and p110δ knockdown cells, tumor progression was significantly lower in p110β- and p110δ-knockdown cell-injected mice compared with scramble cell-injected mice (*P*<0.05); whereas tumor growth observed in p110α- and p110γ-knockdown cell-injected mice was similar to control mice (Figures 2a and b). We speculate that this might be due to markedly decreased tumor cell growth triggered by MM cell adhesion to BM-MSCs, as the adhesion of MM cells to BM-MSCs activates many pathways and has a vital role in MM pathogenesis and disease progression.^12^ We further confirmed that tumor cells showed knockdown for each p110 isoform, as demonstrated *ex vivo* on tumor cells harvested from each cohort of mice (Figure 2c). Mice were followed until the development of hind limb paralysis or death, and Kaplan–Meier analysis was performed showing prolonged survival in all groups except p110α mice (p110β and p110γ, *P*<0.05; p110δ, *P*<0.001; Figure 2d). Despite similar tumor burden observed between p110γ mice and scramble control-injected mice, mice injected with p110γ knockdown cells had improved survival compared with control mice. This might be due to the different extent of tumor involvement of various organs^13^ between the two groups, thus explaining the differences in survival.

Interestingly, our data indicate that p110α is not critical for the survival of MM cells *in vivo*. Unlike most solid tumor malignancies, where PI3KCA (p110α) mutation is the leading cause of activation of this pathway and is the target of many therapeutic agents in development,^3^ there have been no reports of this specific mutations in MM.^6^ Moreover, it was shown that unlike wild-type p110α, overexpression of the wild-type p110β, p110γ and p110δ is sufficient to induce an oncogenic transformation of fibroblasts in cell culture.^14^

In this study, p110β was highly expressed in all MM cell lines, whereas only a minor subset expressed p110δ at the protein level (Figure 1b), which is consistent with a recent report^9^ showing expression of p110β in 38 MM cell lines in comparison to the detectable expression of p110δ in only 4 cell lines. In addition, another study^8^ reported similar findings in cell lines showing lack of p110δ expression in most MM cell lines. Of note, we found discrepancies in p110δ expression in cell lines between our study and prior published studies but our data was confirmed in the Cancer Cell Line Encyclopedia data at the mRNA level (data not shown).^15^ Importantly, Ikeda *et al.*^8^ evaluated p110δ levels in patient samples and detected its expression in all 24 MM patients. This may provide a clinical rationale for targeting p110δ despite the lack of expression of p110δ in MM cell lines.

Overall, our data suggest that, in contrast with solid tumors, MM may be more dependent on PI3K p110β and p110δ and less dependent on PI3Kα, and these may be the focus of drug development in this hematological malignancy.

## Figures and Tables

**Figure 1:**
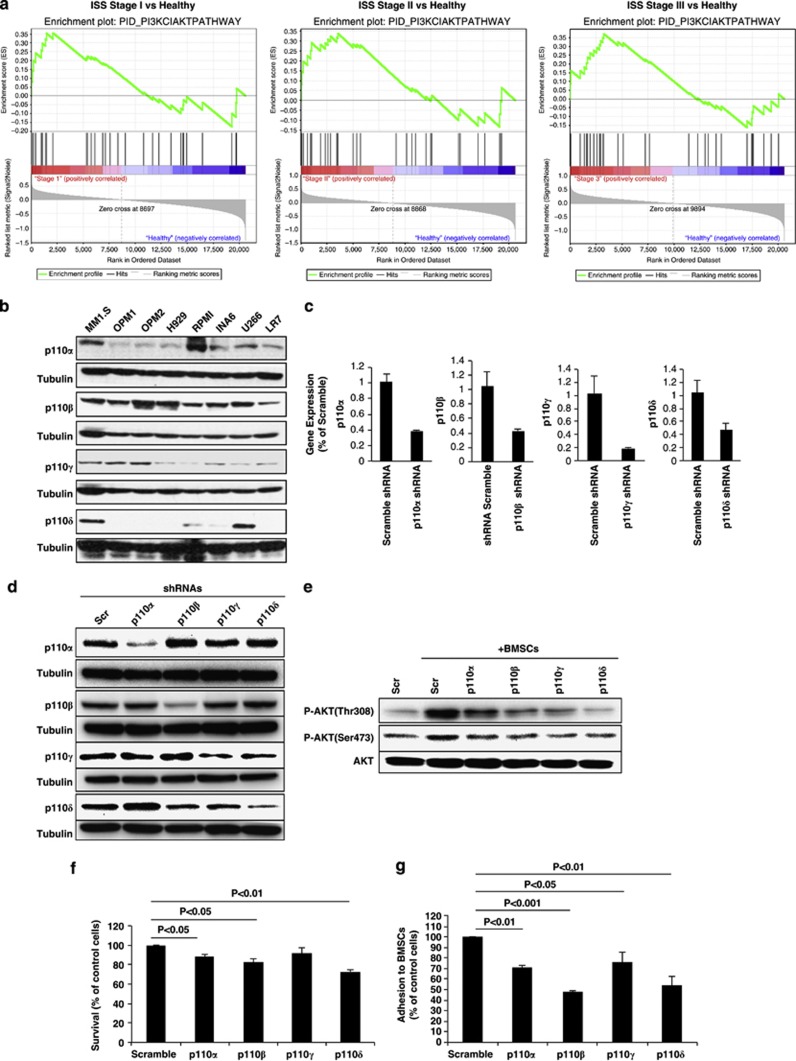
The role of class I PI3K-mediated Akt signaling in MM. (**a**) Gene set enrichment analysis software analyzed functionally related genes in class I-mediated Akt activation with statistically significant enrichment (false-discovery rate *q*-values <0.25; <0.25 is considered significant), using gene-expression data set (GSE24080). Plots show enrichment results for the gene set (left, stage I MM vs normal subjects; middle, stage II MM vs normal subject; right, stage III MM vs normal subjects). (**b**) Baseline expression of the different PI3K isoforms (p110α, β, γ and δ) in MM cell lines was detected by immunoblotting using isoform-specific antibodies. MM tumor cells (MM.1S-GFP^+^/luc^+^) were infected with lentivirus-mediated small hairpin (sh)RNA. Reverse transcription quantitative PCR (**c**) and immunoblotting (**d**) were performed to show infection efficiency and isoform specificity, respectively. Scramble and knockdown tumor cells (p110α, β, γ and δ) were cocultured with BMSCs overnight, and MM cells were then separated from the BMSCs, lysed and whole-cell lysates were subjected to immunoblotting (**e**) with Akt and P-Akt (Thr308 and Ser473), which shows decreased phosphorylation of Akt in knockdown cells. The effects of inhibition of PI3K isoforms by shRNAs on cell survival were assessed by 3-(4,5-dimethylthiazol-2-yl)2-2-diphenyltetrazolium bromide (MTT) assay (**f**). Adhesion assay (**g**) was performed to show the ability of knockdown cells to adhere to BMSCs after 2 h of incubation.

**Figure 2:**
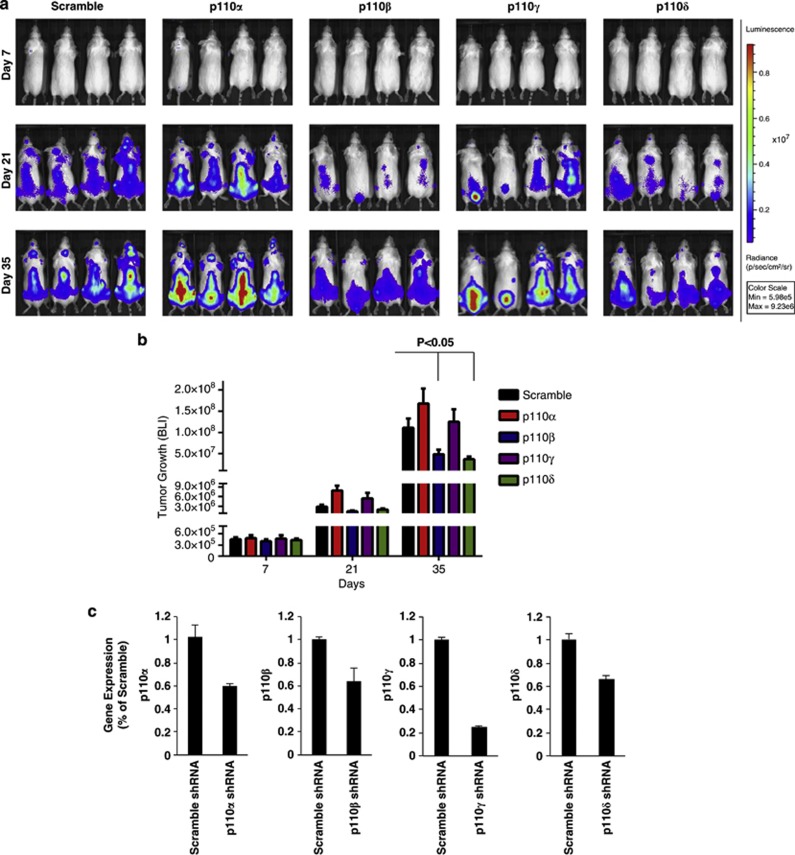

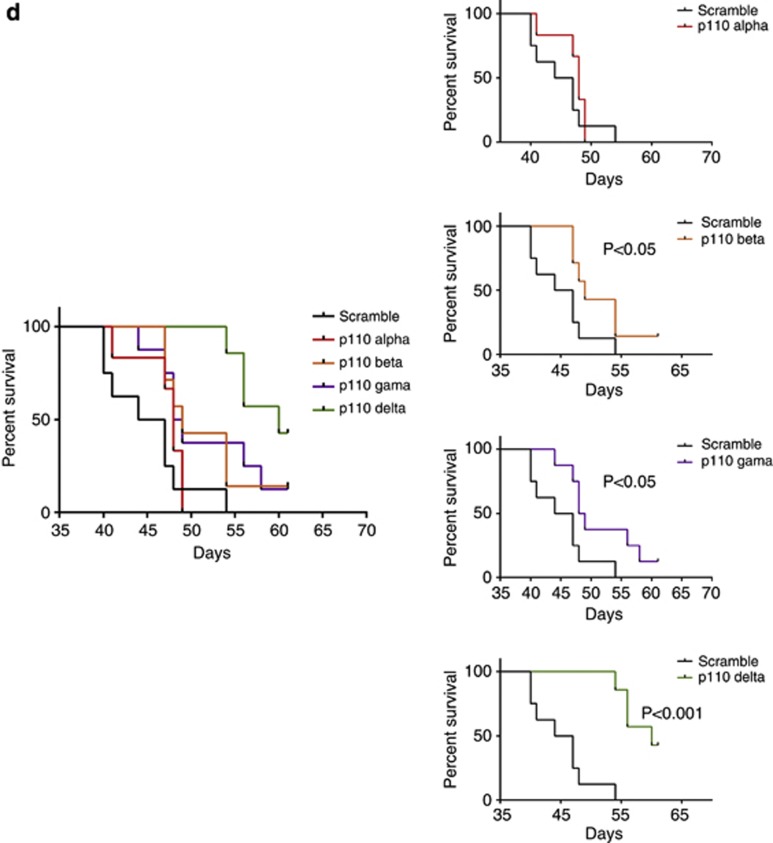
Knockdown of PI3K isoforms regulates tumor progression and survival *in vivo*. MM.1S-GFP^+^/luc^+^ tumor cell lines (Scr, p110α, β, γ and δ) were injected intravenously into SCID-Bg mice and tumor growth was assessed by *in vivo* bioluminescence imaging (BLI). (**a**) Representative BLI of each group in different time points is shown. (**b**) Quantification of BLI signals demonstrated that p110β and δ mice showed significant reduction in tumor growth (*P*<0.05) compared with scramble mice. (**c**) Reverse transcription quantitative PCR was performed on tumor cells that were harvested from hind leg bones of animals by bone marrow flushing. (**d**) Survival of mice was evaluated until complete hind limb paralysis or death using Kaplan–Meier curves. Compared with scramble mice, all groups except p110α showed prolonged survival (p110β and p110γ, *P*<0.05; p110δ, *P*<0.001).

## Notes

IMG is on the advisory board for Onyx, BMS and Celgene, and receives research lab support from Genzyme and BMS. The remaining authors declare no conflict of interest.
