# Supplementary material for: Distinct roles of class I PI3K isoforms in multiple myeloma cell survival and dissemination
Source: Blood Cancer J. 2014 Apr 25;4(4):e204–. doi: 10.1038/bcj.2014.24 (PMC4003418; doi:10.1038/bcj.2014.24)
Supplement: Supplementary Information [file bcj201424x1.doc]

**SUPPLEMENTAL MATERIALS CONTENTS**

**SUPPLEMENTAL METHODS**

**SUPPLEMENTAL FIGURE**

**SUPPLEMENTAL TABLE**

**SUPPLEMENTAL REFERENCES**

**SUPPLEMENTAL METHODS**

**Gene Set Enrichment Analysis (GSEA):**

To determine whether the gene set[^1^](#_ENREF_1) of class I PI3K signaling events mediated by Akt is enriched in International Staging System (ISS) stage I, II and III MM patients compared to healthy subjects (23, 20, 23 and 22 subjects; respectively) in published dataset by Shaughnessy et al (serious number GSE 24080), GSEA was performed following the developer’s protocol (http://www.broad.mit.edu/gsea/).[^2^](#_ENREF_2)

**Cells**

The MM cell lines, OPM1, OPM2, H929, RPMI8226, U266, INA6 and LR7 were kindly provided by Prof Jesús F. San Miguel, University of Salamanca, Salamanca, Spain. MM.1S were purchased from ATCC. All cell lines were cultured at 37^0^ C in RPMI-1640 containing 10% FBS (Sigma Chemical), 2mmol/L glutamine, 100 U/ml penicillin, and 100 µg/ml streptomycin (Gibco). Bone marrow stromal cells (BMSCs) cultures were established as described previously.[^3^](#_ENREF_3) Informed consent was obtained from all patients in accordance with the Declaration of Helsinki for primary MM patient samples.

**Generation of stable shRNA-expressing cell lines**

Lentivirus supernatants of interest were obtained from The RNAi Consortium (TRC). As a control, the shRNA scramble vector was used, which contains a shRNA expression cassette with a target sequence not corresponding to any known mouse, or human genes. The sequence of each shRNA was shown in supplemental table 1. MM.1S-GFP+/luc+ cells were transduced with virus supernatants and Polybrene at 8 μg/ml (Hexadimethrine bromide; Sigma) and selected with puromycin 24 hours after the transduction.

**Animals**

Animal experiments were approved by Dana-Farber Cancer Institute and Massachusetts General Hospital Institutional Animal Care and Use Committees. Female SCID-Bg mice (7-9 weeks of age) were obtained from Taconic and injected with MM cells (Scr, p110α, p110β, p110γ and p110δ). Additional details are provided in detail in the supplemental methods.

To assess the effect of PI3K isoform knockdown on tumor burden in vivo 40 SCID-Bg mice were divided into 5 groups (8 animals/group) and each group injected with MM.1S-GFP+/luc+ cells (5×10^6^) (Scr, p110α, p110β, p110γ and p110δ) via tail vein. Tumor development was monitored once a week by bioluminescent imaging (BLI) for 7 weeks. Survival was evaluated from the first day of treatment until complete paralysis of hind limbs or death (mice that died due to the study procedure during the study were excluded; 2 mice from p110α group, 1 from p110β group and 1 from p110δ group).

**Immunoblotting**

For expression of the different PI3K isoforms, MM cells (MM.1S, OPM1, OPM2, H929, RPMI, INA6, U266 and LR7) and for specificity of isoform specific knockdown scramble and knockdown tumor cells (p110α, β, γ and δ); were cultured, harvested and lysed. For the effect of knockdown of PI3K on PI3K-Akt signaling, scramble and knockdown cells were co-cultured with BMSCs for overnight and tumor cells were then separated from the BMSCs by gentle pipetting and lysed. Proteins were detected by the use of immunoblotting as previously described.[^3^](#_ENREF_3) α-tubulin was used as a loading control. The following mAbs were used for immunoblotting: p110α, p110β, p110γ, p110δ, Akt, P-Akt (Thr308 and Ser473). All mAbs except p110δ, were purchased from Cell Signaling (Technologies Danvers, MA). p110δ was purchased from Santa-Cruz (Santa-Cruz, CA).

**Quantitative reverse transcriptase PCR**

RNA extraction was performed using the RNeasy kit (QIAGEN). RNA was analyzed using an Agilent 2100 bioanalyzer. All PCR reactions were run in triplicate, and mRNA expression, relative to 18S rRNA, was calculated using the 2−ΔΔCt method.[^4^](#_ENREF_4)

**Cell viability test**

Cell growth was assessed by measuring MTT (Chemicon) dye absorbance, as described previously.[^5^](#_ENREF_5)

**Adhesion assays in vitro**

We conducted adhesion assays using BMSCs or fibronectin coated plates, as described previously.[^6^](#_ENREF_6)

**Cell cycle**

MM cells were stained with propidium iodine (PI; Sigma Chemical) and cell cycle was determined using an Epics (Coulter Immunology) flow cytometry as previously described.[^7^](#_ENREF_7)

**Statistical analysis**

Statistical differences between experimental groups were analyzed using Microsoft Office Excel 2007 software using t tests (2-tailed; α 0.05) or ANOVA. P values less than 0.05 were considered significant. Exact P values are provided in the figures. For survival analysis, GraphPad Prism software using Kaplan-Meier curves and log-rank analysis was used.

**Supplementary Figure 1.**

**
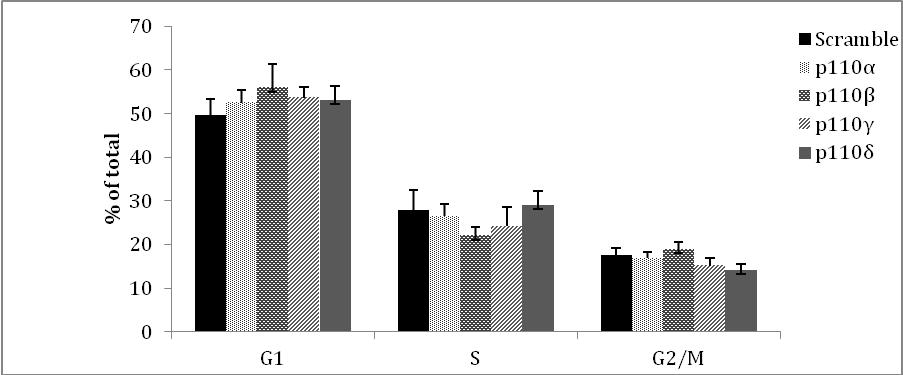
**

**Supplementary Figure1.** **PI3K knockdown has no effect on cell cycle distribution.** Scramble and knockdown tumor cells (p110α, β, γ and δ) were cultured and cell cycle was assessed by PI staining and flow cytometric analysis.

**Supplementary Table 1. shRNA sequences used in this study.**

| Gene | Region | Target Sequence |
| --- | --- | --- |
| Scramble |  | CCGTCATAGCGATAACGAGTT |
| PIK3CA | CDS | GAATTGGAGATCGTCACAATA |
| PIK3CB | CDS | GATTGTGCCCTCTCTAGATTC |
| PIK3CG | 3UTR | GCCTTATCCATTTCCCATTTA |
| PIK3CD | 3UTR | GATCTTTCTCTCTGACTATAC |

**Reference list**

**1. Schaefer CF, Anthony K, Krupa S, Buchoff J, Day M, Hannay T*, et al.* PID: the Pathway Interaction Database. *Nucleic acids research* 2009 Jan; 37(Database issue): D674-679.**

**2. Subramanian A, Tamayo P, Mootha VK, Mukherjee S, Ebert BL, Gillette MA*, et al.* Gene set enrichment analysis: a knowledge-based approach for interpreting genome-wide expression profiles. *Proceedings of the National Academy of Sciences of the United States of America* 2005 Oct 25; 102(43): 15545-15550.**

**3. Leleu X, Jia X, Runnels J, Ngo HT, Moreau AS, Farag M*, et al.* The Akt pathway regulates survival and homing in Waldenstrom macroglobulinemia. *Blood* 2007 Dec 15; 110(13): 4417-4426.**

**4. Livak KJ, Schmittgen TD. Analysis of relative gene expression data using real-time quantitative PCR and the 2(-Delta Delta C(T)) Method. *Methods* 2001 Dec; 25(4): 402-408.**

**5. Roccaro AM, Sacco A, Husu EN, Pitsillides C, Vesole S, Azab AK*, et al.* Dual targeting of the PI3K/Akt/mTOR pathway as an antitumor strategy in Waldenstrom macroglobulinemia. *Blood* 2010 Jan 21; 115(3): 559-569.**

**6. Azab AK, Azab F, Blotta S, Pitsillides CM, Thompson B, Runnels JM*, et al.* RhoA and Rac1 GTPases play major and differential roles in stromal cell-derived factor-1-induced cell adhesion and chemotaxis in multiple myeloma. *Blood* 2009 Jul 16; 114(3): 619-629.**

**7. Roccaro AM, Sacco A, Thompson B, Leleu X, Azab AK, Azab F*, et al.* MicroRNAs 15a and 16 regulate tumor proliferation in multiple myeloma. *Blood* 2009 Jun 25; 113(26): 6669-6680.**
